# Eu-Doped Citrate-Coated Carbonated Apatite Luminescent Nanoprobes for Drug Delivery

**DOI:** 10.3390/nano10020199

**Published:** 2020-01-23

**Authors:** Ylenia Jabalera, Francesca Oltolina, Maria Prat, Concepcion Jimenez-Lopez, Jorge F. Fernández-Sánchez, Duane Choquesillo-Lazarte, Jaime Gómez-Morales

**Affiliations:** 1 Departamento de Microbiología, Facultad de Ciencias, Universidad de Granada, Campus de Fuentenueva s/n, 18002 Granada, Spain; yjabalera@ugr.es (Y.J.); francesca.oltolina@med.uniupo.it (F.O.); cjl@ugr.es (C.J.-L.); 2 Dipartimento di Scienze della Salute, Università del Piemonte Orientale “A. Avogadro”, Via Solaroli 17, 28100 Novara, Italy; maria.prat@med.uniupo.it; 3 Department of Analytical Chemistry, Faculty of Sciences, University of Granada, Avda. Fuentenueva s/n, 18071 Granada, Spain; jffernan@ugr.es; 4 Laboratorio de Estudios Cristalográficos, IACT-CSIC-Universidad de Granada, Avda. Las Palmeras, 4, 18100 Armilla, Spain; duane.choquesillo@csic.es

**Keywords:** Eu-doped citrate-nanoapatites, Doxorubicin, adsorption isotherm, desorption, luminescence, cytotoxicity

## Abstract

In the field of Nanomedicine, there is an increasing demand for new inorganic nanophosphors with low cytotoxicity and efficient loading-release ability of drugs for applications in bioimaging and drug delivery. This work assesses the potentiality of matured Eu-doped citrate-coated carbonated apatite nanoparticles to be used as theranostic platforms, for bioimaging, as luminescent nanoprobes, and for drug delivery applications, using Doxorubicin as a model drug. The drug adsorption isotherm fits the Langmuir–Freundlich (LF) model, showing that the Eu:cit-cAp nanoparticles can carry a maximum of 0.29 ± 0.02 mg Doxo mg Eu:cit-cAp^−1^ (*Q_max_*). The affinity constant K_FL_ for this binding is 44 ± 2 mL mg^−1^, and the cooperativity coefficient r is 6 ± 1. The nanoparticle suspensions presented charge reversion from negative to positive after loading with Doxo as revealed by the ζ-potential versus pH characterization. The release of drug from the loaded nanoparticles was found to be strongly pH-dependent, being around 5 wt % at physiological pH 7.4 and 20 wt % at pH 5, in experiments lasting 24 h. Luminescence spectroscopic measurements of Doxo-loaded nanoparticles revealed the increase of luminescence with a decrease in the amount of adsorbed Doxo, due to the so-called inner filter effect. The nanoparticles free of Doxo were cytocompatible when interacted with two human cell lines derived respectively from a gastric carcinoma (GTL-16), and a hepatocarcinoma (Huh7), while Doxo-loaded nanoparticles displayed significant toxicity in a dose-dependent relationship. Therefore, the new nanoassemblies might have a dual function, as nanoprobes in bioimaging by detecting the fate of the nanoparticles in biological environments, and for monitoring the delivery of the drug in such environments, by measuring the rise of the luminescence provided by the desorption of Doxo.

## 1. Introduction

For more than 10–15 years, nanoparticles (NPs) are offering new opportunities in different fields, including medicine, namely because they can act as multifunctional platforms for both diagnostic and therapeutic applications [1,2,3]. For the former, biosensing and bioimaging can be approached by the use of luminescent nanoparticles that can provide the required fluorescent contrast [4]. Fluorescent inorganic and organic nanoparticles including quantum dots, silica, gold, conjugated polymers, organic dyes and structures labeled with organic dyes and monoclonal antibodies have been largely investigated [5,6,7,8,9,10,11]. Among them, and in the context of bioimaging, some organic fluorescent dyes-based nanomaterials have been reported to present favorable characteristics compared to those of inorganic nanoparticles such as higher biodegradability, biocompatibility and lower toxicity [12], especially when compared to quantum dots, which are somehow cytotoxic and show photoblinking [5,13]. In spite of the systems already developed, there is still room for new ones with the goal of improving efficiency and lowering toxicity.

In this context, lanthanides-doped nanoapatites represent a new class of nanophosphors with improved properties. Indeed, they present color tuning depending on the doped lanthanide ion, sharp emission peaks, a long fluorescence lifetime, a high quantum yield, and good resistance to photobleaching from environment [11,14,15,16,17]. Apatite is an easily dopable structure that exhibits favorable properties for bioimaging applications such as the absence of toxicity, biodegradability, biocompatibility, and low or nonexistent inflammatory and immunity response. It is stable at physiological conditions (~7.4, T ~37 °C), while it can partially dissolve at acidic pHs, such as those found at tumor microenvironments or within lysosomes. Moreover, apatite can be functionalized with different molecules, which can be released in response to local stimuli, e.g., a change in pH [18,19,20]. Because of these special features, lanthanide-doped apatite nanoparticles are excellent candidates as theranostic platforms, i.e., for bioimaging and for drug delivery applications [21,22,23,24,25,26]. This would also allow a simultaneous tracing of the carried molecule while being delivered.

Recently, we have prepared biomimetic (bone-like) citrate-coated europium doped carbonated apatite (Eu:cit-cAp) nanoparticles as well as citrate-coated calcium doped europium phosphate monohydrate (Ca:cit-EuPO_4_·H_2_O, rhabdophane type) nanophosphors [27,28] at different cation doping concentrations and maturation times by the citrate-based thermal decomplexing method [29,30]. In the former case, the technique allows the preparation of the single doped apatitic phase with nanosized dimensions only when the Eu^3+^ doping concentrations are ≤0.01 M. The presence of carbonate on the apatite structure and the citrate coating on the nanoparticle surface (both features found in bone nanoapatites) and Eu^3+^ substituting Ca^2+^ in the structure enhanced the stability of the Eu:cit-cAp suspensions at physiological pH, as shown by the measurements of ζ-potential versus pH [27]. In addition, the nanoparticles presented high luminescence intensity, a long luminescence lifetime (in the order of the millisecond), and were non-cytotoxic, thus suggesting they could be used in bioimaging applications. However, the ability of these Eu:cit-cAp nanoprobes as nanocarriers for drugs, as well as the monitoring of release of the drug by luminescence spectroscopy, has never been explored. 

Doxorubicin (Doxo), a potent chemotherapeutic molecule [31] whose administration involves significant nonspecific side effects [32,33], can be used as a model drug to test these abilities. In aqueous solutions Doxo, which consists of three planar and aromatic hydroxyanthraquinonic rings [33], forms dimers with antiparallel configuration [34], in which the −NH_3_^+^ groups are arranged in opposite directions [35]. The adsorption of Doxo on hydrophilic nanocarriers takes place by electrostatic interactions at the solid–solution interface and, therefore, it can be governed by pH changes [36].

In this work, we have addressed the proof of concept of the potential use of Eu:cit-cAp to be used as a dual platform for bioimaging and drug carrier. As Eu:cit-cAp was already demonstrated to be a good nanophosphor [27], we have now studied its drug delivery ability, using Doxo as a model. Drug loading, release as a function of pH, stability of the aqueous suspensions, effect of the loaded drug on nanoparticle luminescence properties, as well as the cytotoxicity of the drug-loaded nanoparticles in in vitro assays against two human cell lines derived respectively from a gastric carcinoma (GTL-16), and a hepatocarcinoma (Huh7), were analyzed.

## 2. Materials and Methods 

### 2.1. Reagents

Europium chloride hexahydrate (EuCl_3_·6H_2_O, ACS Reagent, 99.9% pure), calcium chloride dihydrate (CaCl_2_·2H_2_O, Bioxtra, 99.0% pure), sodium citrate tribasic dihydrate (Na_3_(cit)·2H_2_O, with cit = citrate = C_6_H_5_O_7_, ACS reagent, ≥99.0% pure) and disodium hydrogenphosphate (Na_2_HPO_4_, ACS reagent, ≥99.0% pure) were provided by Sigma-Aldrich (St. Louis, MO, US). Sodium carbonate monohydrate (Na_2_CO_3_·H_2_O, ACS reagent, 99.5% pure) and hydrochloric acid (HCl, ACS reagent, 37 wt % in H_2_O) were provided by Merck (Darmstadt, Germany) and Panreac (Darmstadt, Germany), respectively. All solutions were prepared with ultrapure water (0.22 µS, 25 °C, Milli-Q, Millipore, Burlington, MA, US). Doxorubicin hydrochloride (Doxo, C_27_H_29_NO_11_·HCl, molecular weight without HCl 543.52 g/mol) was purchased from Sigma-Aldrich. 

### 2.2. Preparation and Characterization of Eu:cit-cAp Nanocarriers

The preparation of the nanocarriers was performed following the citrate-based thermal decomplexing method reported in reference [27], using a Eu^3+^doping concentration 0.01 M, a maturation time of 15 days, and the same post-synthesis treatment. The resulting Eu-doped sample was characterized by powder X-ray diffraction (XRD), scanning and transmission electron microscopies (SEM and TEM), Fourier transform infrared (FTIR) spectroscopy, dynamic light scattering (DLS) and electrophoretic mobility. 

XRD data were collected with a Bruker D8 Advance Vario diffractometer (Bruker GmbH, Karlsruhe, Germany) using Cu Kα1 radiation (1.5406 Å). SEM observations and energy dispersive X-ray spectroscopy (EDX) were performed with a variable pressure Zeiss SUPRA40VP scanning electron microscope (VPSEM, (Carl Zeiss, Jena, Germany) provided of a large X-Max 50 mm area detector for EDX microanalysis. The analysis of Eu, P and Ca were determined by EDX averaging 7–10 measurements in 10 different Eu-apatite particles. TEM observations and selected area electron diffraction (SAED) were performed with a Carl Zeiss Libra 120 microscope (at 80 kV, Carl Zeiss, Jena, Germany). Powder samples were dispersed in absolute ethanol (≥99.8%) by ultrasonication. Then, droplets of the slurry were deposited on formvar coated copper microgrids prior to observation. The FTIR spectrum was recorded with a Perkin Elmer Spectrum One FTIR spectrometer (Perkin-Elmer, Beaconsfield, UK). The pellet was prepared by pressing with a hydraulic pump (10 t into 13 mm diameter discs) a mixture of ~1 mg of sample and ~100 mg of anhydrous KBr. The particle size distribution and electrophoretic mobility (transformed to ζ-potential values) as a function of pH were analyzed with a Malvern Zetasizer Nano ZS analyzer (Malvern Instruments Ltd, Malvern, UK,) using disposable polystyrene cuvettes containing the particles suspended in deionized water (0.5 mg/mL) at 25 °C. For the measurements of both the electrophoretic mobility and the particle size distribution versus pH, the MPT-2 autotitrator (Malvern, UK) connected to the analyzer was used, introducing diluted HCl or NaOH solutions (0.25 and 0.1 M, respectively) as titration agents. 

### 2.3. Doxo Adsorption and Release Using Eu:cit-cAp Nanocarriers

The time at which equilibrium was attained for the adsorption of Doxo onto the nanoparticles was before determined by means of a kinetic study. Five milligrams of nanoparticles were mixed with 1 mL of aqueous Doxo (1 mg/mL), and the samples were incubated at 37 °C for different periods of time within the interval 0 to 48 h. The samples were stirred continuously at 150 rpm in the dark to prevent the photodegradation of Doxo [37]. Then, nanoparticles were separated from the solution by centrifugation at 10,000 rpm for 5 min. Pellets were carefully rinsed three times with 1 mL of ultrapure water and the four supernatants obtained were collected and measured by UV-Vis spectroscopy at 480 nm to determine the non-adsorbed Doxo, which would provide an indirect determination of the Doxo in equilibrium (*C_e_*) and the amount of adsorbed Doxo per mass unit of adsorbent (*Q*). The molar absorptivity of Doxo in solution was determined from the slope of a standard calibration straight line as 24.7 ± 0.5 mg mL^−1^ cm^−1^ (see Figure S1 and Table S1 in Supplementary Materials (SM)). Experiments were replicated three times. The standard deviation of the absorbance measurements was used to estimate the error in the concentration of Doxo in the supernatant ([Doxo]_sn_). The kinetics data were fitted to the Lagergren’s equation (Equation (S1) of SM), in which *Q* is the amount of Doxo on the nanoparticle surface and τ is the time needed to reach approximately a 63% of the drug loading capacity (*Q_max_*) [20]. 

Once the time at which the adsorption of Doxo onto the nanoparticles reached equilibrium was known, experiments to determine the adsorption isotherm were performed by fixing the reaction time to 24 h to ensure the equilibrium of the system. Five mg of nanoparticles were mixed with 1 mL of Doxo at different concentrations (ranging from 0.01 to 1.5 mg mL^−1^) and then the resulting suspensions were incubated at 37 °C. At least 10 independent experiments and three replicas per each experiment were performed to plot the adsorption isotherm. The data were fitted to the Langmuir–Freundlich (LF) model, by using Origin Pro 8 ((Washington, WA, US)), described by Equation (1). The LF model considers that the adsorption energy is heterogeneous and takes into account cooperativity effects. In this equation, *Q* is the amount of adsorbed drug per amount of nanoparticles, *Q_max_* is the drug loading capacity, *C_e_* is the equilibrium concentration of drug in the supernatant, *K_LF_* is the LF affinity constant, and r is the cooperativity coefficient. Values of r > 1 indicate a positive cooperativity, while values of r < 1 indicate a negative cooperativity [20,36,38,39]:

(1) $$Q=\frac{Q_{max}{\left(K_{LF}C_{e}\right)}^{r}}{1+{\left(K_{LF}C_{e}\right)}^{r}}.$$

The stability of the nanoassembly at physiological pH (pH 7.4) and the potential drug release at acidic pH (pH 5.0) were also evaluated. To measure the stability, the functionalized nanoparticles (5 mg) were washed twice with ultrapure water and resuspended in HEPES (10 mM, 10 mL, pH 7.4). To measure Doxo release at acidic pH, identical experiments were performed by suspending the nanoassemblies in sodium citrate/citric acid solution (10 mL, pH 5.0). Suspensions were incubated at 37 °C, 150 rpm for different time intervals up to 48 h, and were then separated from supernatants by centrifugation at 10,000 rpm for 5 min. An aliquot of each supernatant was collected, analyzed by UV-Vis spectroscopy and returned to its initial suspension. The release efficiency (*D_R_*) was defined by Equation (2), as the ratio between the amount of released molecules at a certain time t (*Q*_(*t*)_) and the drug loading capacity, *Q_max_* [20,36]:

(2) $$D_{R}=\frac{Q_{\left(t\right)}}{Q_{max}}\times 100.$$

### 2.4. Luminescence Spectroscopy

Excitation and emission spectra of the aqueous suspensions of the nanoparticles (~0.5 mg/mL) were recorded using a Cary Eclipse Varian Fluorescence Spectrophotometer (Varian Australia, Mulgrave, Australia). The following instrumental conditions were used: λ_exc/em_ = 394/614 nm, delay time (t_d_) = 120 µs, gate time (t_g_) = 5 ms, excitation and emission slits = 10 nm, detector voltage = 800 V. For determining the lifetime of the aqueous suspensions of the nanoparticles (0.5 mg/mL), λ_exc/em_ = 394/614 nm, delay time (t_d_) = 100 µs, gate time (t_g_) = 0.01 ms, excitation and emission slits = 10 nm, detector voltage = 780 V. 

Excitation and emission spectra of Doxo in solutions were recorded with the same instrument but measuring in fluorescence mode using the following instrumental conditions: λ_exc/em_ = 500/590 nm, excitation and emission slits = 10 nm, detector voltage = 550 V.

### 2.5. Cytotoxicity Tests 

GTL-16 cells, a human gastric cell carcinoma [40] (12 × 10^3^/0.4 cm^2^ microwell), and Huh7 cells, a human hepatocellular carcinoma [41] (6 × 10^3^/0.4 cm^2^ microwell), were incubated for 24 h and afterward, different concentrations of Doxo and Doxo-loaded nanoparticles, ranging from 0.01 to 100 µg mL^−1^, were added in 100 µL. After 3 days of incubation, cell viability was evaluated by the 3-(4,5-Dimethylthiazol-2-yl)-2,5-diphenyltetrazolium bromide) (MTT, Sigma-Aldrich, St. Louis, Mo, USA) colorimetric assay. Briefly, 20 µL of MTT solution (5 mg mL^−1^ in a PBS solution) were added to each well. The plate was then incubated at 37 °C for 3 h. After the removal of the solution, 0.2 M HCl acidified isopropanol was added for dissolution of formazan crystals. Optical density was measured in a multi-well reader (2030 Multilabel Reader Victor TM X4, PerkinElmer, Waltham, MA, USA) at 570 nm. Experiments were performed 4 times using triplicates for each sample. One-way ANOVA with Dunnett’s post-test was performed using GraphPad Prism version 4.00 for Windows, GraphPad Software (GraphPad Prism, San Diego, CA, USA). Optical images of the cells at a Leica ICC50 HD microscopy (Munich, Germany) were acquired after 3 days incubation, before the treatment with MTT.

## 3. Results and Discussion 

### 3.1. Physicochemical and Morphological Characteristics of Eu:cit-cAp Nanocarriers

**Figure 1:**
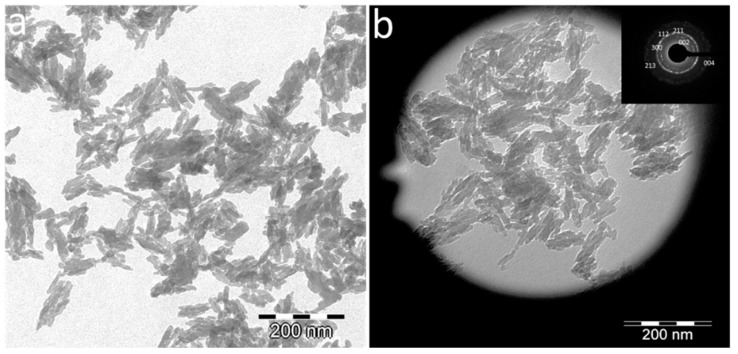
(**a**) TEM micrograph of Eu:cit-cAp nanocarriers prepared by thermal decomplexing of Ca/Eu/citrate/phosphate/carbonate solutions [27]; (**b**) selected area of the microgrid showing the nanoparticles for electrons diffraction, and SAED pattern (inset) showing the main reflections of the apatitic phase, i.e., 002, 211, 112, 300, 213, 004.

The nanocarriers displayed plate-shape elongated morphologies with average length (L = 40 ± 8 nm) and width (W = 17 ± 4 nm) (Figure 1a), a Ca/P ratio = 1.54 ± 0.04 and wt % Eu = 5.4 ± 1.2. The SAED pattern (Figure 1b, inset) shows rings corresponding to the main reflections of the apatite phase, i.e., 002, 211, 112, 300, 213 and 004.

**Figure 2:**
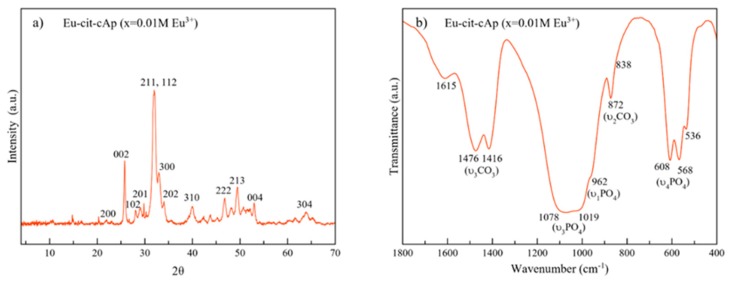
(**a**) XRD pattern of Eu:cit-cAp prepared by thermal decomplexing of Ca/Eu/citrate/phosphate/carbonate solutions [27]; (**b**) FTIR spectrum of Eu:cit-cAp.

The XRD pattern (Figure 2a) displays the main reflections of the apatite phase (PDF 00-055-0592), i.e., at 2θ = 25.87° (002), the triplet at 31.77°, 32.19° and 32.90° (211,112 and 300, respectively), the reflections at 34.03° and 39.81° (202 and 310) and other minor peaks in the 2θ range from 40–65°.

The FTIR spectrum (Figure 2b) in the region from 400 to 1800 cm^−1^ shows a broad band at 1000–1100 cm^−1^ corresponding to the asymmetric stretching mode of PO_4_^3−^ groups (υ_3_PO_4_). The shoulder at ~962 cm^−1^ is ascribed to the symmetric stretching (υ_1_PO4) and those bands at ~608 and 568 cm^−1^ correspond to the bending mode (υ_4_PO_4_) of PO_4_^3−^ groups [16]. The signal at 536 cm^−1^ is assigned to surface HPO_4_^2−^ ions, which points to the biomimetic nature of these apatites [42]. The presence of carbonate (CO_3_^2−^) bands at ~1416 cm^−1^ and 1476 cm^−1^ (υ_3_CO_3_ mode), and at 872 cm^−1^ (υ_2_CO_3_) confirms the presence of CO_3_^2−^ ions doping the structure, most of them replacing PO_4_^3−^ lattice ions (B-type) [29]. Besides apatitic vibrational contributions, the band at ~1600 cm^−1^ is assigned to the antisymmetric stretching frequency of the –COO^−^ groups of the citrate ions [43].

### 3.2. Doxo Adsorption and Release

**Figure 3:**
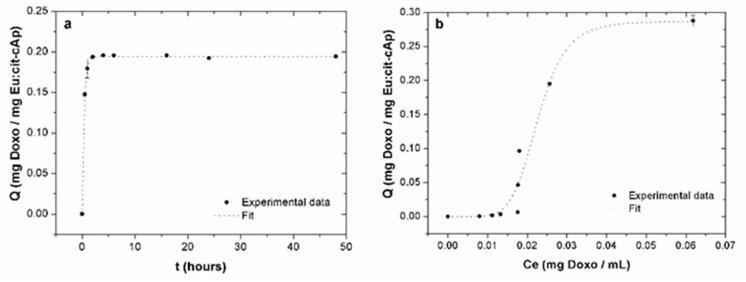
(**a**) adsorption kinetics of Doxo on Eu:cit-cAp nanocrystals. Dotted blue line represents the nonlinear fitting of the experimental data; (**b**) adsorption isotherm of Doxo on Eu:cit-cAp nanocarriers. The dotted blue line represents the nonlinear fitting of experimental data using the Langmuir−Freundlich equation.

According to the calculations based on the Lagergren’s equation, τ was 0.36 ± 0.07 h (R^2^ = 0.99934). Therefore, these kinetic data of Doxo adsorption on Eu:cit-cAp nanocarriers over time show that ~2 h was the time needed for this system to attain equilibrium (Figure 3a).

The adsorption isotherm shows that the amount of adsorbed drug per unit mass of nanocrystals (*Q*) was nonlinear (Figure 3b), the initial increase of *Q* being slow, then exponential and finally stabilizing at a *Q_max_* of 0.28 ± 0.02 mg Doxo mg Eu:cit-cAp^−1^. These experimental data show a nice fit (R^2^ = 0.94597) with the Langmuir–Freundlich (LF) model [44,45] which was somehow expected considering the surface energetic heterogeneities at the different apatite crystal faces. The value for the cooperativity coefficient (r) was of 6 ± 1 (Table 1), which indicates cooperation between the already bound Doxo molecules and the new ones to bind [46]. The LF affinity constant (*K_LF_*) was of 44 ± 2 mL mg^−1^ (Table 1), revealing that adsorbed Doxo molecules are interacting with the substrate besides interacting between themselves, lowering the adsorption energy.

**Figure 4:**
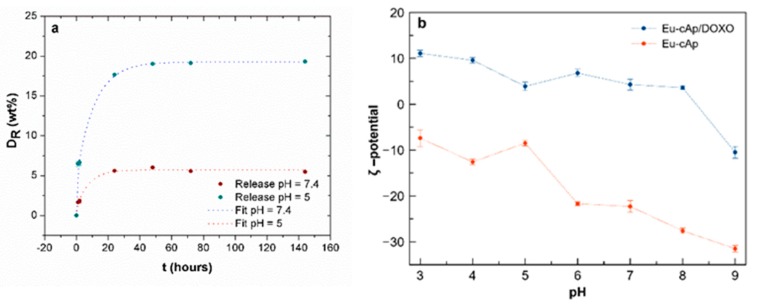
(**a**) kinetics of Doxo release from Eu:cit-cAp/Doxo at pH 7.4 and pH 5.0; (**b**) evolution of ζ–potential of Eu:cit-cAp nanocarriers and Eu:cit-cAp/Doxo at pHs from 3 to 9.

The release of Doxo from the loaded Eu:cit-cAp nanoparticles was found to be strongly pH- dependent (Figure 4a), being much higher at pH 5.0 than at pH 7.4. The Doxo released at physiological pH (pH 7.4) was practically negligible, with *D_R_* values at 24 h ≤5 wt % of the initially adsorbed Doxo. These data show the stability of the nanoassembly at physiological pH values. However, the drug was released more efficiently at acidic pH values (*D_R_* ~20 wt % of the Doxo loaded on nanoparticles) within the first 24 h. 

The results show that Eu:cit-cAp nanocarriers can be functionalized with Doxo, carrying 0.28 mg of drug per mg of Eu:cit-cAp (*Q_max_*). The *Q_max_* value obtained in the present work is lower than the values obtained for the coupling of the same drug on undoped apatite nanoparticles prepared by the citrate based decomplexing method. In this context, for example, the adsorption of Doxo on the undoped cit-Ap and cit-cAp nanoparticles was 0.41 ± 0.06 and 0.44 ± 0.02 mg Doxo mg apatite^-1^ respectively [20]. Nevertheless, the present results reveal that these luminescent nanoprobes could also be used for drug delivery applications. 

An important characteristic of the colloidal Eu:cit-cAp suspensions regarding its usefulness as drug nanocarriers and luminescent probes is the behavior of ζ-potential vs. the pH of their aqueous suspensions, which influences the aggregation of the nanoparticles. The plot of ζ-potential vs pH of the unloaded nanocarriers (Figure 4b, red line) shows that this parameter decreases from −7.4 to −33.5 mV in the pH range from 3 to 9, being −8.5 mV at pH 5 and −22.3 mV at pH 7, therefore allowing the preparation of stable suspensions. In addition, the highly negative ζ-potential values at physiological or higher pHs benefit individual non-aggregated particle loading.

When loaded with Doxo, a reversion of the surface charge of the nanoparticles gives rise to slightly positive ζ-potential values, with variations from +12 mV to +5 mV in the pH range from 3 to 8, being +5 mV at both pHs 5 and 7 (Figure 4b, blue line). These small variations indicate that, after loading with Doxo, the pH has little influence on the ζ-potential, and thus it does not alter the aggregation state of the suspensions. This is reflected in the cumulative volume undersize distributions of the nanoparticle suspensions in the pH range from 4 to 9 (Figure S2) in which the loaded nanoparticles display almost similar size distributions (Figure S2b) while the unloaded ones behave differently (Figure S2a). The loading mechanism is thus determined by two complementary interactions. At physiological pH, the Doxo molecules form dimers in aqueous solutions, exposing the antiparallel configuration [35]. In these loading conditions, the pH is lower than the pKa of Doxo, which determines that their amino groups are protonated. These positively charged −NH_3_^+^ groups allow the electrostatic interaction with the negatively charged free –COO^−^ groups of adsorbed citrate and with the negative >PO_4_^δ−^, >CO_3_^δ−^, and >OH^δ−^ surface species present on the nanoparticles. In relation to the other loading mechanism, the high cooperativity coefficient r obtained in the LF model (r > 1) proves the strong positive cooperativity between the Doxo molecules during the adsorption process. This mode of interaction was previously found in Doxo adsorption on apatite nanocrystals [36,47]. The free −NH_3_^+^ groups pointing outward toward the solution are likely the ones that are responsible for the charge reversion reflected in the positive ζ-potentials values. 

The stability of the nanoparticles functionalized with Doxo and the release of Doxo from nanoparticles are relevant points to be considered for the potential clinical application of nanoparticles. When injected in vivo, nanoparticles would be subjected to different pHs: (1) physiological pH in bloodstream (~7.4); (2) acidic pH (~6) in the tumor environment; and (3) acidic pH (~5) in the endosome–lysosome compartment [48]. In this context, the ideal drug delivery system based on pH-response effect should retain the loaded drug in the bloodstream for a long enough time until they can reach the targeted tumor site, and, once there, it should release most of the carried drug. The previous study on cit-cAp nanocarriers loaded with Doxo revealed that the nanoparticles were uptaken by GTL-16 cancer cells via endocytotic mechanism [20] and were able to carry inside them their bound Doxo. The results of the present work show that Eu:cit-cAp/Doxo nanoassemblies are endowed with the suitable properties for their application as a drug delivery system, since a negligible amount of Doxo release is expected in the bloodstream (thus reducing its adverse side effects) until the nanoparticles reach the target tumor (acidic environment).

### 3.3. Luminescence Properties

Europium (III) and terbium (III) form highly fluorescent chelates with many different organic ligands that emit sensitized fluorescence, emitting the transferred energy as narrow bands, with a long Stokes shift (over 250 nm) and a long fluorescence decay time (up to 1 ms) [49]. This long luminescence lifetime allows the use of adequate delay (t_d_) and gate (t_g_) times to reduce the luminescence background and, therefore, to increase the signal-to-noise ratio.

**Figure 5:**
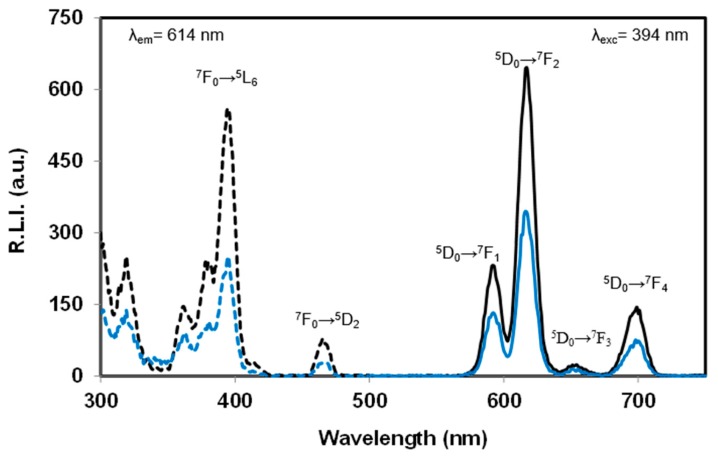
Excitation (dashed line) and emission (solid line) spectra of Eu:cit-cAp nanocarriers free of Doxo (black color) and loaded with 0.037 mg Doxo/mg Eu:cit-cAp (blue color) suspended in water at 25 °C and pH = 7.4. Slit-widths_exc/em_ = 10/10 nm, td = 120 µs, t_g_ = 5 ms, detector voltage 800 V. λ_exc_ = 394 nm; λ_em_ = 614 nm.

The luminescence properties of Eu:cit-cAp nanocarriers free and loaded with Doxo suspended in water at several pHs are depicted in Figure 5, Figures S3 and S4 (uncorrected excitation and emission spectra) and Figures S5 and S6 (luminescence decay curves); for each case, the decay profile was analyzed as a single exponential component (R.L.I.=A·e−tτ+C), where τ is the luminescence lifetime. The determination of the amount of Doxo loaded on the nanocarriers was carried out by measuring the intrinsic fluorescence emission of Doxo in the supernatant after incubation (see Figure S7). 

The observed excitation and emission spectra of the suspended particles free and loaded with Doxo were very similar, thus indicating that functionalization with Doxo does not affect the chemical composition of the adsorbent particles. The excitation wavelengths were 320, 364, 382, 394, and 463 nm, which correspond to the Eu(III) ion transitions ^7^F_0_→^5^H_6_, ^7^F_0_→^5^D_4_, ^7^F_0_→^5^L_7_, ^7^F_0_→^5^L_6_ and ^7^F_0_→^5^D_2_, while the emission wavelengths were 590, 614, 652 and 697 nm, which correspond to the Eu(III) ion transitions ^5^D_0_→^7^F_1_, ^5^D_0_→^7^F_2_, ^5^D_0_→^7^F_3_ and ^5^D_0_→7F_4_, respectively [50]. 

The hypersensitive transition (^5^D_0_→^7^F_2_, 614 nm) provided the highest relative luminescence intensity. This finding agrees with the literature because this emission dominates the spectrum for nanosized particles [51]. Therefore, the optimum excitation and emission wavelengths of the doped nanoparticles were 394 nm and 614 nm, respectively. 

Concerning the variation of τ versus pH, it can be deduced that pH does not affect the luminescence lifetime. However, it is observed that the loading of Doxo on the Eu:cit-cAp particles decreases the lifetime. In the pH range from 5.0 to 7.4, luminescence lifetime of the particles is around 1060 µs, whereas the luminescence lifetime of the Doxo loaded particles is almost 520 µs. This phenomenon is also observed in the luminescence emission (see Figure 5). Thus, the adsorption of Doxo on the nanoparticles quenches the sensitized luminescence signal. 

In general, there are three different types of quenching processes that can occur: static, dynamic and apparent [52]. The static quenching implies the formation of a non-luminescent ground state complex between the luminophore and the quencher, whereas dynamic or collisional quenching occurs when the quencher diffuses to the luminescent specie during the lifetime of its excited state and nonradiatively deactivates that state. Both quenching processes are adequately described by the Stern–Volmer equation (Equation (3)) [53]:

(3) $$\frac{I_{0}}{I}=1+k\left[Q\right],$$

where *I*_0_ and *I* correspond to the luminescence emission in the absence and presence of the quencher, respectively, [*Q*] is the quencher concentration, and *k* corresponds to the quenching constant. For static quenching, *k* corresponds to the association constant for complex formation while the *k* in dynamic corresponds to the Stern–Volmer quenching constant, which can be defined as *k_q_*·*τ_0_*, where *k_q_* is the bimolecular quenching constant and *τ_0_* is the lifetime of the luminophore in the absence of quencher. In general, static and dynamic quenching can be distinguished by their different dependence on temperature and viscosity, or preferably by lifetime measurements. For static quenching *τ_0_*/*τ* = 1, the luminescence lifetime is thus not affected by the presence of the quencher; in contrast, for dynamic quenching, *τ_0_*/*τ* = *I*_0_/*I* [54].

The apparent quenching is not a quenching process at all but is rather due to an attenuation of the excitation beam and/or adsorption of emitted radiation by an excess concentration of luminophore or by the presence of an additional absorbing specie in the media. This phenomenon is more commonly known as the “inner filter effect” [52,55,56,57,58]. 

**Figure 6:**
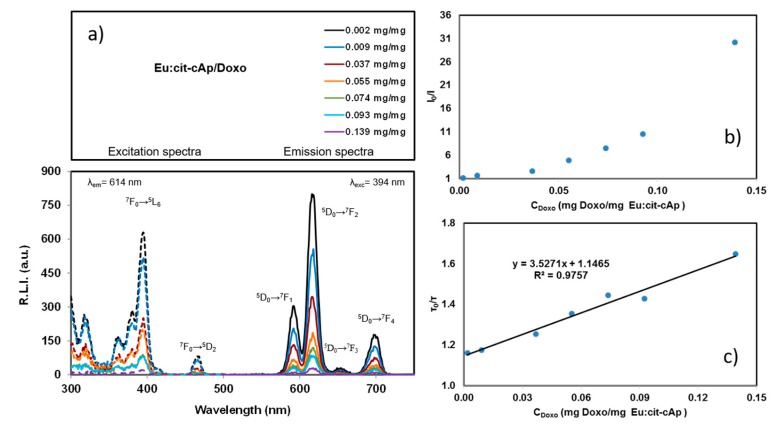
(**a**) excitation (dashed line) and emission (solid line) spectra of Eu:cit-cAp nanoparticles loaded with varying amounts of Doxo, in aqueous suspensions at 25 °C and pH 7.4; (**b**) Stern–Volmer plot in intensity; and (**c**) Stern–Volmer plot in lifetime. λ_exc/em_ = 394/614 nm, slit-widths_exc/em_ = 10/10 nm, td = 120 µs, t_g_ = 5 ms, detector voltage 800 v.

Figure 6 shows the excitation and emission spectra of the Eu:cit-cAp nanoparticles containing different amounts of Doxo (see Figure 6a). In addition, it shows the Stern–Volmer Plots *I_0_*/*I* vs. [Doxo] (see Figure 6b) and *τ_0_*/*τ* vs. [Doxo] (see Figure 6c). 

Figure 6b shows an upward curvature, concave towards the *y*-axis, which is characteristic of the combination of different quenching processes. SM (see Figure S8) and Figure 6c show that the lifetime of the Eu:cit-cAp/Doxo particles is affected by the immobilized amount of Doxo (C*_Doxo_*). Therefore, it is possible to deduce that dynamic quenching occurs. On the other hand, the plot of the apparent quenching constant (*k_app_*) versus C*_Doxo_* does not provide a linear relationship (see SM, Figure S9) indicating that static quenching is ruled out [54]. 

**Figure 7:**
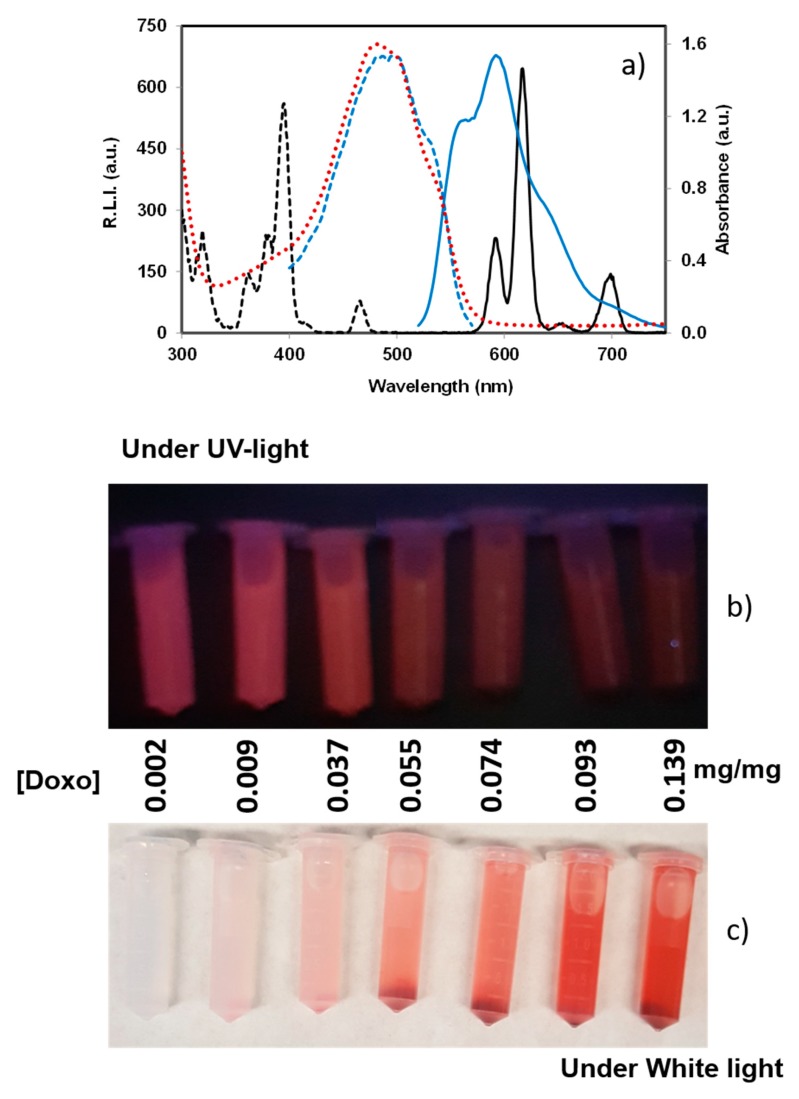
(**a**) excitation (black dashed line) and emission (black solid line) spectra of Eu:cit-cAp particles and excitation (blue dashed line), emission (blue solid line), and adsorption (red dotted line) of 0.01 mg/mL Doxo solution at the optima instrumental conditions (see Experimental Section). The pictures show: (**b**) the decrease of luminescence and (**c**) the increase of the light adsorption when the amount of immobilized Doxo increased on Eu:cit-cAp nanoparticles.

Finally, Figure 7 shows the excitation (black dashed line) and emission (black solid line) spectra of Eu:cit-cAp (black color) and the excitation (blue dashed line), emission (blue solid line) and absorption (red dotted line) spectra of Eu:cit-cAp/Doxo in aqueous suspension. It shows an overlap between the absorption spectra of Doxo and the excitation spectra of the particles. Thus, when Doxo is immobilized on the Eu:cit-cAp particles, it can adsorb the excitation light and, therefore, the number of excited Eu(III) atoms decreased, thus providing a decrease in the luminescence emission by the inner filter effect. 

To sum up, the decrease of the luminescence emission of the Eu:cit-cAp/Doxo particles might be attributed to a combination of dynamic quenching and the inner filter effects of immobilized Doxo. 

Due to their luminescent properties, the new nano-assemblies might be used for applications in bioimaging by detecting the fate of the nanoparticles in biological environments, as well as for monitoring the delivery of the drug by measuring the rise of the luminescence provided by the desorption of Doxo. This dual function represents a great advantage with respect to previous undoped apatite nanoparticles in view of its potential theranostic applications.

### 3.4. Cytotoxicity

**Figure 8:**
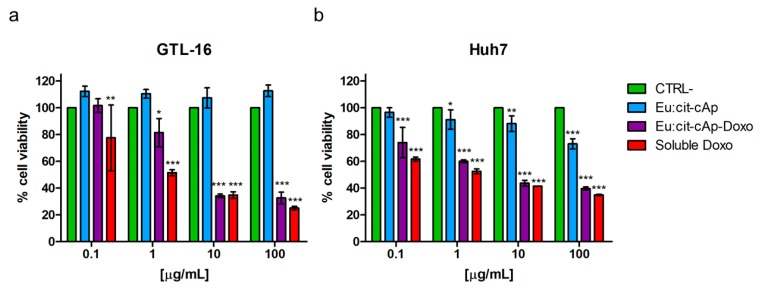
Results of the MTT assays performed with Eu-citAp-NPs functionalized with Doxo on GTL-16 (**a**) and Huh7 (**b**) cells. Data are expressed as cell viability compared to the untreated controls (CTRL-) at the same time points. On abscissae, the amounts of nanoparticles are reported and, for each point, the same amount of Doxo that was loaded on nanoparticles was used also as a soluble drug. Data represent means ± SD of four independent experiments performed in triplicate, and statistical analyses were carried on using one-way ANOVA, with a Bonferroni comparison test. For statistical analysis, all data were compared to untreated samples.

**Figure 9:**
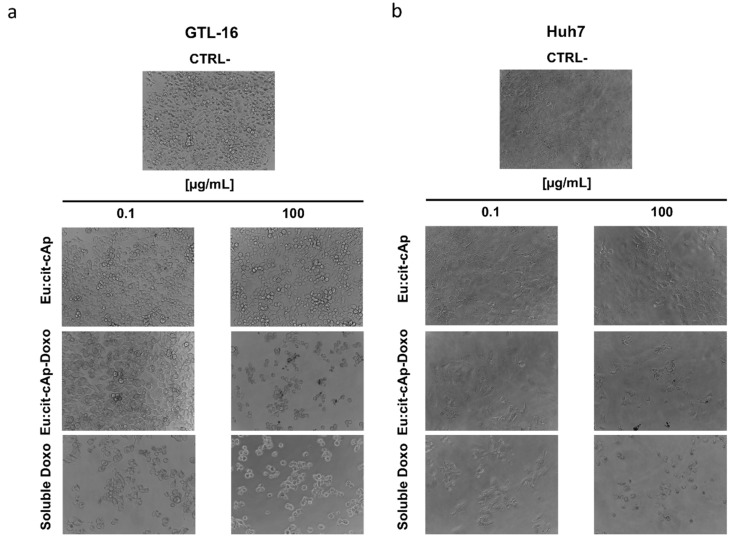
Representative photographs of the GTL-16 (**a**) and Huh7 (**b**) cells treated or untreated (ctrl-) with not functionalized and Doxo-functionalized Eu-citAp-NPs and soluble Doxo at two different concentrations. The same amount of Doxo that was loaded on nanoparticles was used also as soluble drug (240×).

The biological effects of the Eu:cit-cAp/Doxo nanoparticles were tested on two human tumor cell lines: GTL-16 and Huh7 cells. As expected, the Doxo-free nanoparticles did not display significant cytotoxicity, since only at their higher concentration was the viability of Huh7 cells decreased, but it was always higher than 70% (Figure 8b), which is the cut-off indicated by ISO 10993–5:2009 [59]. Doxo-loaded NPs displayed a significant toxicity on both cell lines in a dose-dependent relationship, with Huh7 cells appearing to be more sensitive in general to NPs (Figure 8a,b). Representative images of the cells after the treatments and of control untreated cells are reported in Figure 9, which are in agreement with the data of the MTT assay. 

When compared to the toxicity exerted by soluble Doxo, these Doxo-loaded nanoparticles were found to be less toxic, at least at the lower nanoparticles concentrations tested. Similar findings were reported also for other Doxo-loaded nanoparticles prepared with different carriers [20,60,61,62,63] and do not represent a drawback of these compounds. Indeed, although they have a lower cytotoxic activity than soluble Doxo, they can circulate in the blood stream for longer periods, and thus should exert more efficient passive targeting of the tumor through the so-called enhanced permeability and retention (EPR) effect typical of tumor immature vasculature [64].

## 4. Conclusions

In the present study, we have developed multifunctional nanoparticles that possess the ability to serve as both drug-delivery vehicles and optical bioimaging probes for eventual in vivo applications. The cytocompatible Eu:cit-cAp nanoparticles were shown to act as efficient and smart Doxo loading/release nanocarriers. Doxo adsorption on nanoparticles fitted the LF model, with a maximum drug-loading capacity of 0.29 mg Doxo/mg nanoparticles, while drug desorption was pH-responsive, reaching a maximum of about 25% of the loaded drug at pH 5, the last one simulating the acidic pH in the endosome-lysosome compartment of cancer cells. Luminescence intensity was raised linearly with the release of the drug, enabling the monitoring of its desorption. These Eu:cit-cAp/Doxo nano-assemblies exerted cytotoxicity on two human tumor cell lines in a dose-dependent relationship. The properties of these luminescent Doxo-loaded nanoparticles make them promising candidates to be used as a theranostic platform in applications for bioimaging and drug delivery in a cancerous environment, whose efficacy could be even improved by further functionalization with tumor targeting probes in the context of personalized medicine. 

## Figures and Tables

**Table 1:** Adsorption parameters calculated from nonlinear fitting of experimental data according to the Langmuir−Freundlich equation.

Parameter	Doxo/Eu:cit-cAp	R^2^
*K_LF_* [mL mg^−1^]	44 ± 2	
*Q_max_* [mg Doxo mg Eu:cit-cAp^−1^]	0.28 ± 0.02	0.94597
r	6 ± 1	

## Supplementary Materials

The following are available online at https://www.mdpi.com/2079-4991/10/2/199/s1, Figure S1: Standard calibration straight line Doxo on Eu:cit-cAp nanocrystals. Table S1: Kinetics parameters obtained from the lineal fitting of the experimental data. Equation S1: Lagergren’s equation. Figure S2. Cumulative volume undersize distribution of Eu-citcAp particles in the pH range from 3 to 9 (a) before and (b) after loading with Doxo. Figure S3: (a) excitation (dashed line) and emission (solid line) spectra of Eu:cit-cAp nanoparticles suspended in water at 25 °C at several pHs and (b) the effect of the pH on the luminescence emission of these particles. Figure S4: (a) excitation (dashed line) and emission (solid line) spectra of Eu:cit-cAp nanoparticles loaded with 0.139 mg Doxo/mg Eu:cit-cAp suspended in water at 25 °C at several pHs, and (b) the effect of the pH on the luminescence emission of these particles. Figure S5: Luminescence decay curve of Eu:cit-cAp nanoparticles suspended in water at 25 °C at several pHs. Figure S6: Luminescence decay curve of Eu:cit-cAp nanoparticles loaded with 0.139 mg Doxo/mg Eu:cit-cAp suspended in water at 25 °C at several pHs. Figure S7: (a,b) calibration curve of Doxo in water, and (c) determination of the absorbed Doxo on the Eu:cit-cAp nanoparticles versus the equilibrium Doxo concentration. Figure S8: Luminescence decay curve of Eu:cit-cAp/Doxo nanoparticles with varying concentration of Doxo suspended in HEPES buffer at pH = 7.4 and 25 °C. Figure S9: Variation of the apparent quenching constant with the concentration of the quencher.
