# Supplementary material for: Eu-Doped Citrate-Coated Carbonated Apatite Luminescent Nanoprobes for Drug Delivery
Source: Nanomaterials (Basel). 2020 Jan 23;10(2):199. doi: 10.3390/nano10020199 (PMC7074876; doi:10.3390/nano10020199)
Supplement: Supplementary file 1 [file nanomaterials-10-00199-s001.pdf]

Supplementary Materials

Eu-Doped Citrate-Coated Carbonated Apatite Luminescent Nanoprobes for Drug Delivery

Ylenia Jabalera [1], Francesca Oltolina [1,2], Maria Prat [2], Concepcion Jimenez-Lopez [1], Jorge F. Fernández-Sánchez [3], Duane Choquesillo-Lazarte [4] and Jaime Gómez-Morales [4,*]

[1] Departamento de Microbiología, Facultad de Ciencias, Universidad de Granada, Campus de Fuentenueva s/n, 18002 Granada, Spain; yjabalera@correo.ugr.es (Y.J.); cjl@ugr.es (C.J.-L.)

[2] Dipartimento di Scienze della Salute, Università del Piemonte Orientale "A. Avogadro", Via Solaroli 17, 28100 Novara, Italy; francesca.oltolina@med.uniupo.it (F.O); maria.prat@med.uniupo.it (M.P.)

[3] Department of Analytical Chemistry, Faculty of Sciences, University of Granada, Avda. Fuentenueva s/n, 18071 Granada, Spain; jffernan@ugr.es (J.F.-S.)

[4] Laboratorio de Estudios Cristalográficos, IACT-CSIC-Universidad de Granada, Avda. Las Palmeras, 4, 18100 Armilla, Spain; duane.choquesillo@csic.es (S.C.-L.)

* Correspondence: jaime@lec.csic.es (J.G.-M.); Tel.: +34-958-230000 (ext. 190203)

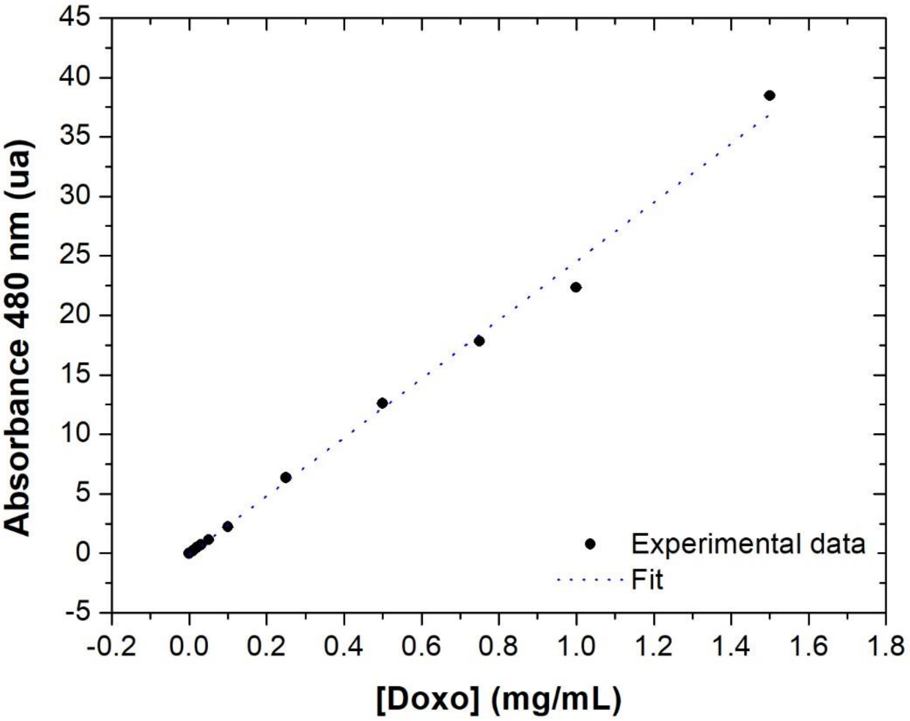

Figure S1. Standard calibration straight line Doxo on Eu:cit-cAp nanocrystals. Dotted blue line represents the lineal fitting of the experimental data.

Table S1. Kinetics parameters obtained from the lineal fitting of the experimental data.

Parameter		R^2
Intercept [ua]	-0.1 ± 0.3	0.99424
Slope [mL mg^{-1}]	24.7 ± 0.6	

Equation S1:

$$Q(t) = Q_{max}\,(1\text{-}e^{\,(-t/\tau)}) \qquad \text{(Lagergren's equation)}$$

Q is the amount of drug adsorbed on the nanoparticle surface and τ the time needed to reach approximately a 63% of Q_{max}

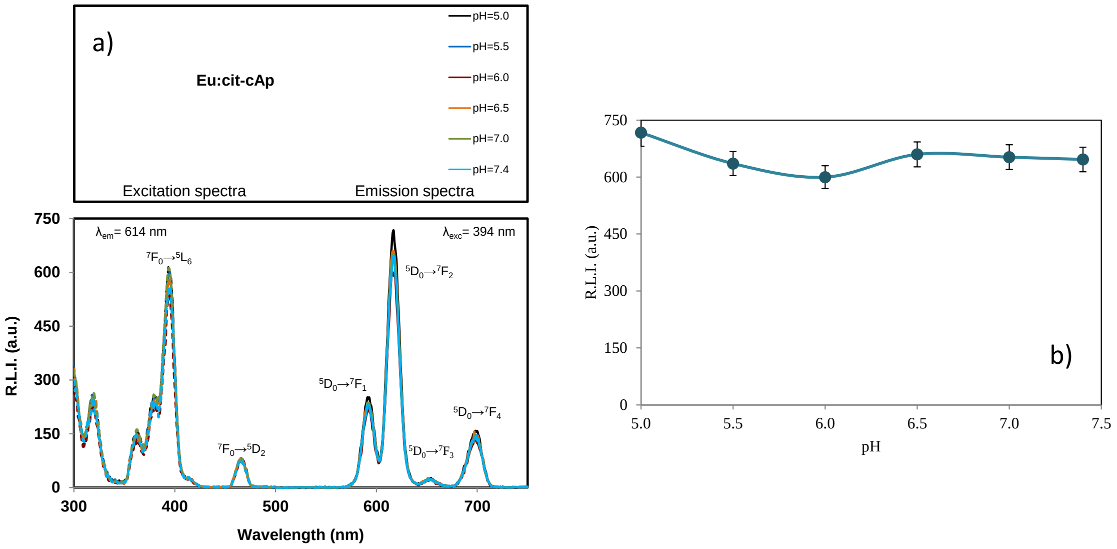

Figure S2. (**a**) excitation (dashed line) and emission (solid line) spectra of Eu:cit-cAp nanoparticles suspended in water at 25 °C at several pHs and (**b**) the effect of the pH on the luminescence emission of these particles; slit-widths$_{exc/em}$ = 10/10 nm, t_d = 120 μs, t_g = 5 ms, detector voltage 800v.

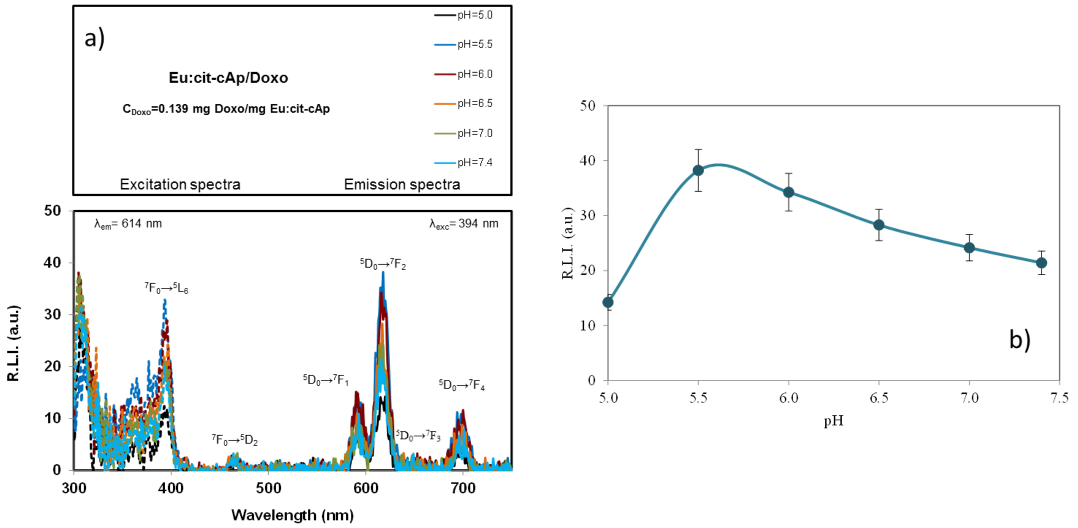

Figure S3. (**a**) excitation (dashed line) and emission (solid line) spectra of Eu:cit-cAp nanoparticles loaded with 0.139 mg Doxo/mg Eu:cit-cAp suspended in water at 25 °C at several pHs, and (**b**) the effect of the pH on the luminescence emission of these particles; slit-widths$_{exc/em}$ = 10/10 nm, t_d = 120 μs, t_g = 5 ms, detector voltage 800v.

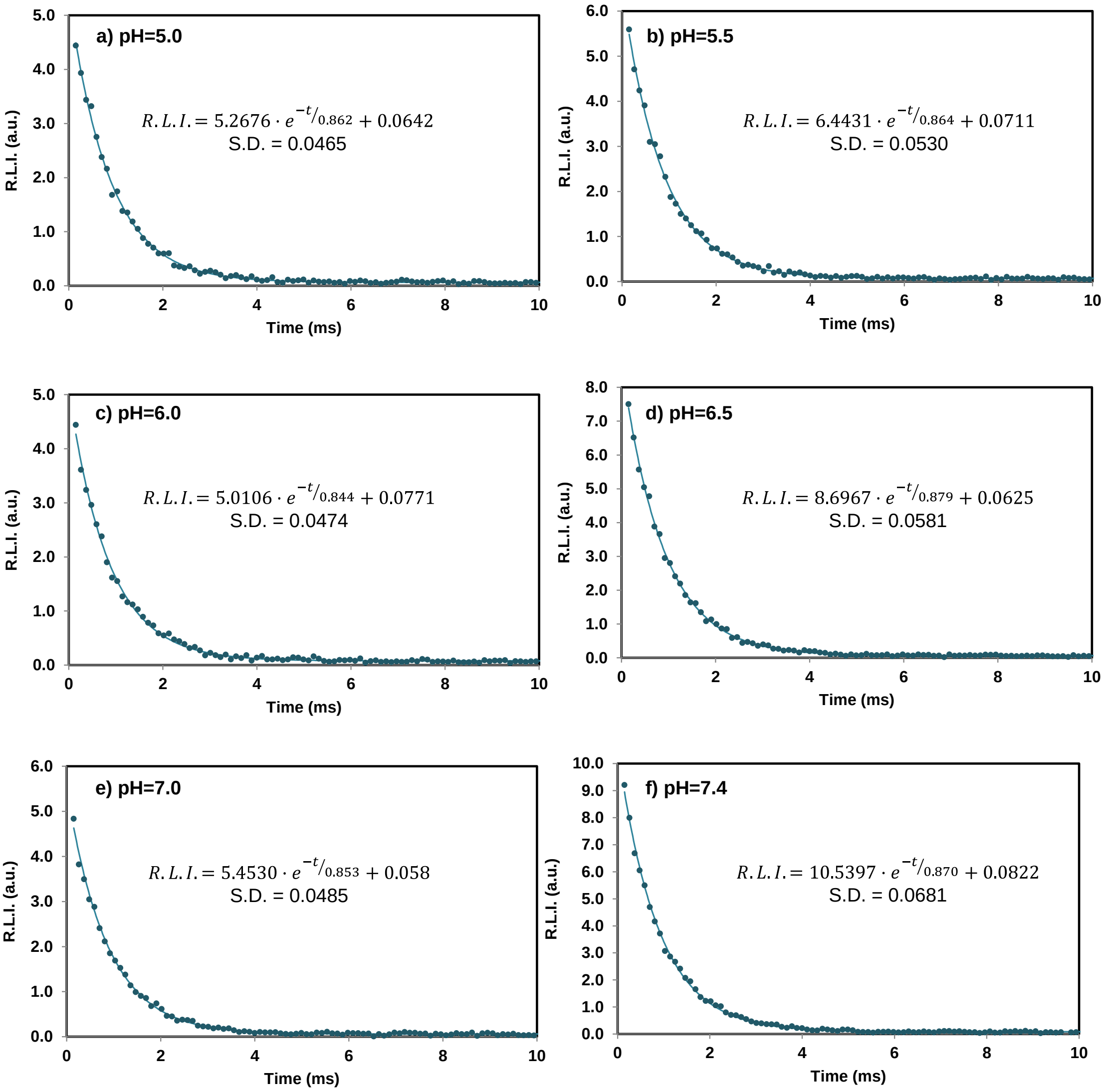

Figure S4. luminescence decay curve of Eu:cit-cAp nanoparticles suspended in water at 25 °C at several pHs, $\lambda_{exc/em}$=394/618 nm, slit-widths$_{exc/em}$ = 10/10 nm, and detector voltage = 780 V. Circles correspond to experimental data and lines to the fitting equation.

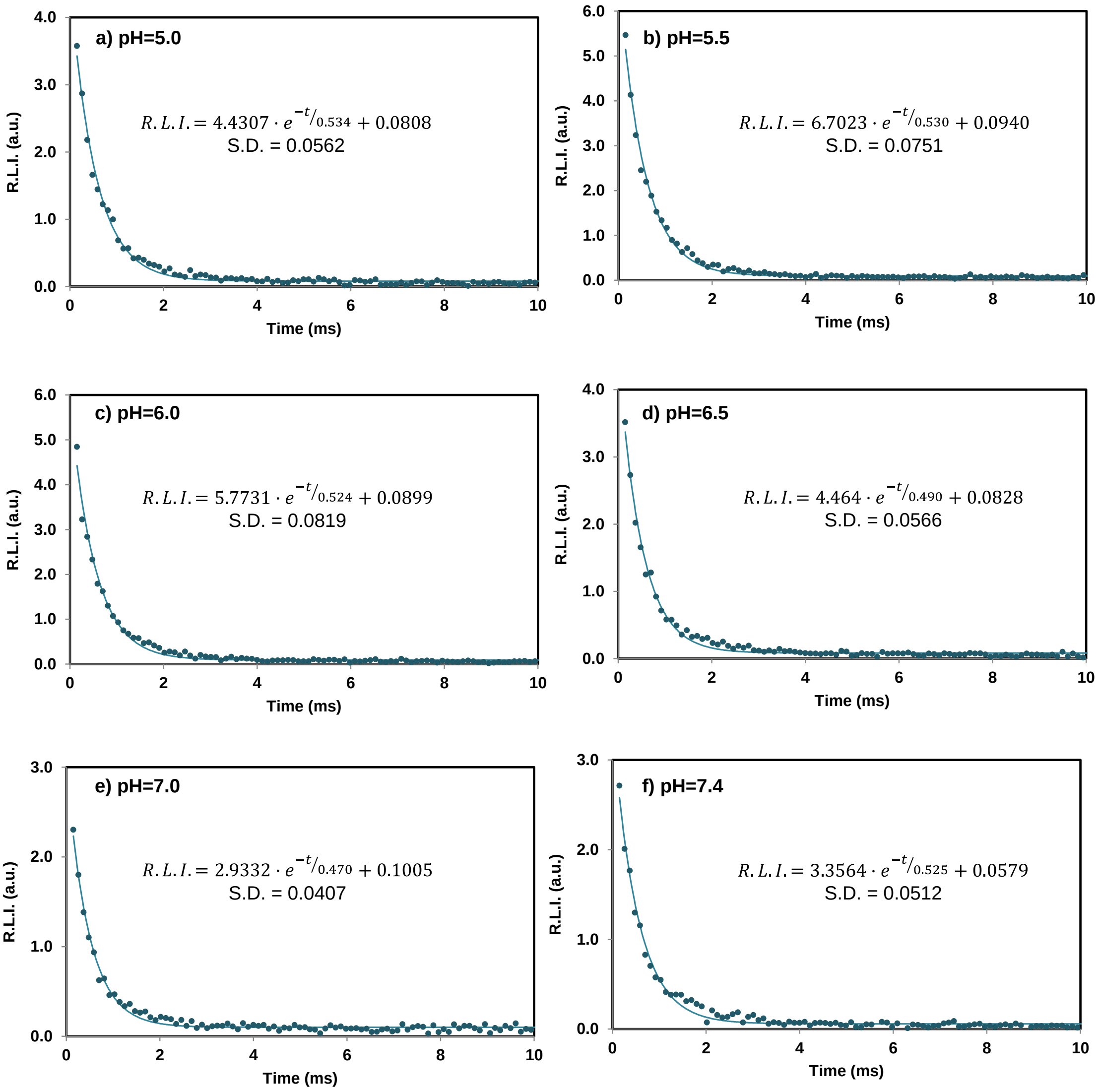

Figure S5. luminescence decay curve of Eu:cit-cAp nanoparticles loaded with 0.139 mg Doxo/mg Eu:cit-cAp suspended in water at 25 °C at several pHs. $\lambda_{exc/em} = 394/614$ nm, slit-widths$_{exc/em} = 10/10$ nm, and detector voltage = 780 V; circles correspond to experimental data and lines to the fitting equation.

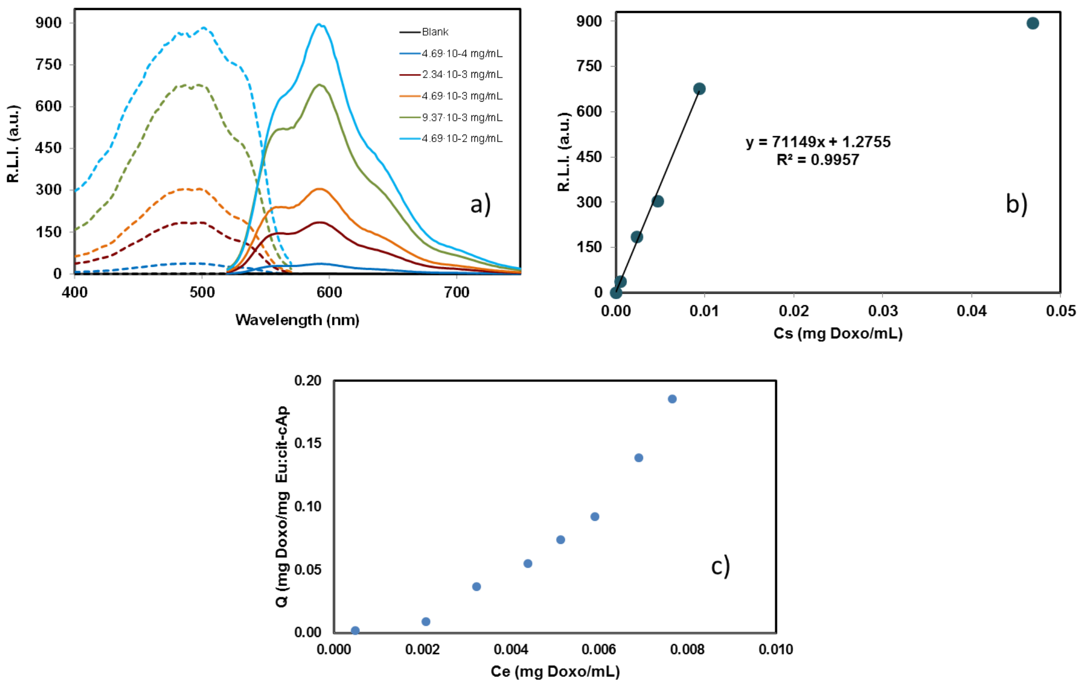

Figure S6. (**a**, **b**) calibration curve of Doxo in water, and (**c**) determination of the absorbed Doxo on the Eu:cit-cAp nanoparticles versus the equilibrium Doxo concentration.

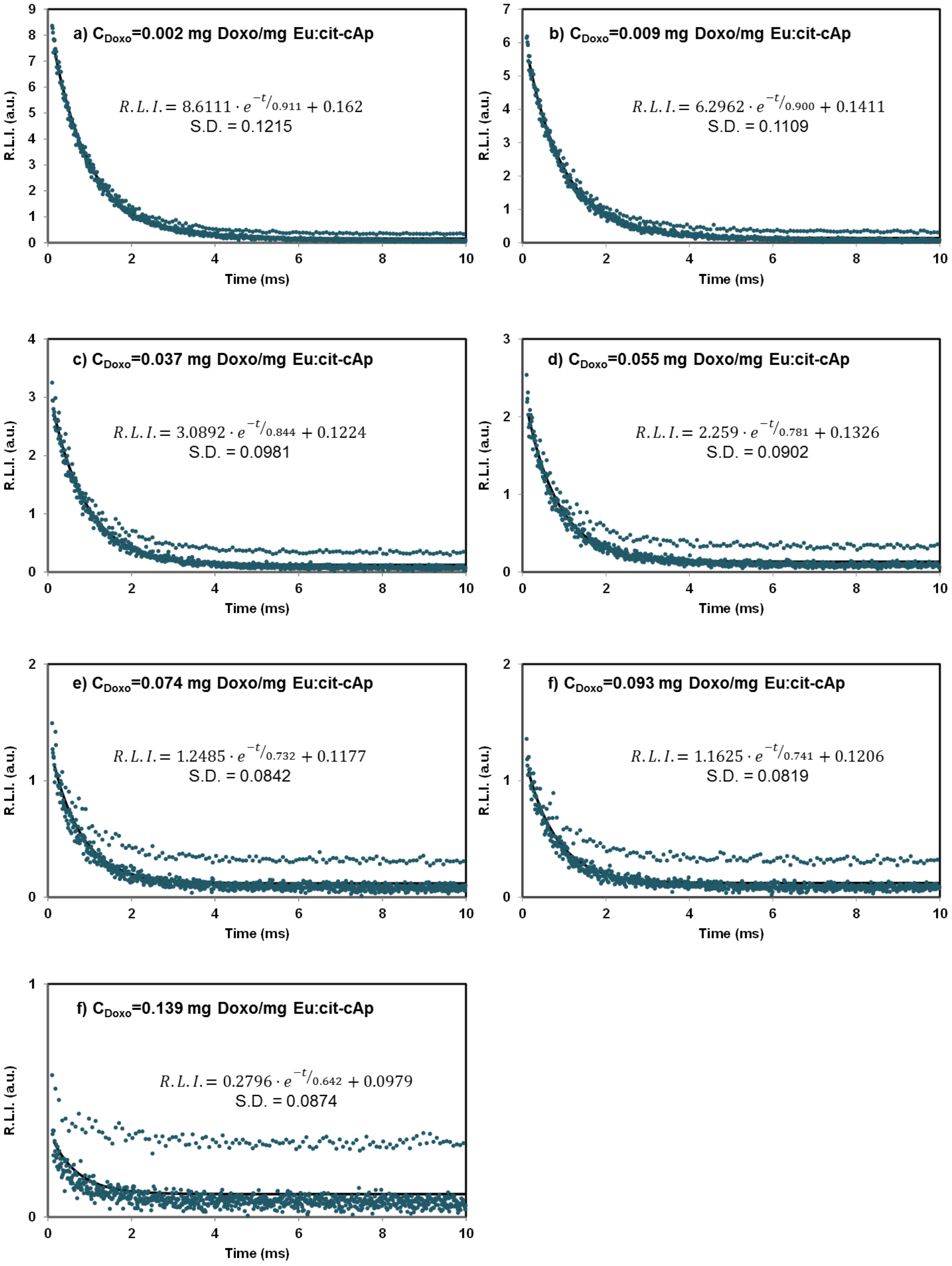

Figure S7. luminescence decay curve of Eu:cit-cAp/Doxo nanoparticles with varying concentration of Doxo suspended in HEPES buffer at pH=7.4 and 25ºC; $\lambda_{exc/em}$ = 394/614 nm, slit-widths$_{exc/em}$ = 10/10 nm, and detector voltage = 780 V; circles correspond to experimental data and lines to the fitting equation.

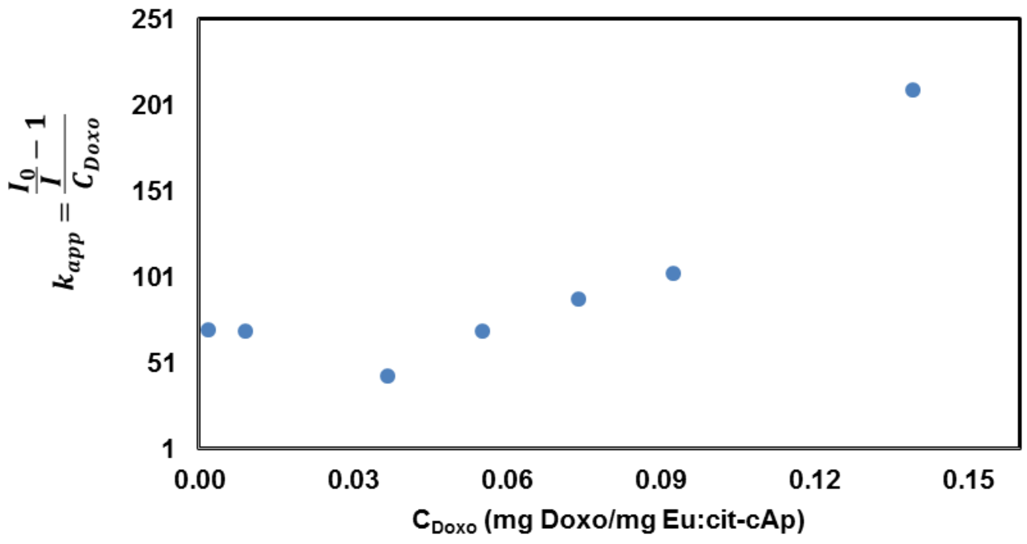

Figure S8. Variation of the apparent quenching constant with the concentration of the quencher.
